# Co-expression of double-stranded RNA and viral capsid protein in the novel engineered *Escherichia coli* DualX-B15(DE3) strain

**DOI:** 10.1186/s12866-021-02148-8

**Published:** 2021-03-23

**Authors:** Kitti Wuthisathid, Thawatchai Chaijarasphong, Charoonroj Chotwiwatthanakun, Monsicha Somrit, Kallaya Sritunyalucksana, Ornchuma Itsathitphaisarn

**Affiliations:** 1 Department of Biochemistry, Faculty of Science, Mahidol University, Bangkok, 10400 Thailand; 2 Center of Excellence for Shrimp Molecular Biology and Biotechnology (Centex Shrimp), Faculty of Science, Mahidol University, Bangkok, 10400 Thailand; 3 Department of Biotechnology, Faculty of Science, Mahidol University, Bangkok, 10400 Thailand; 4 Nakhonsawan Campus, Mahidol University, Nakhonsawan, 60130 Thailand; 5 Department of Anatomy, Faculty of Science, Mahidol University, Bangkok, 10400 Thailand; 6 Aquatic Animal Health Research Team (AQHT), National Center for Genetic Engineering and Biotechnology (BIOTEC), National Science and Technology Development Agency (NSTDA), Yothi Office, Bangkok, 10400 Thailand

**Keywords:** *Escherichia coli*, dsRNA, Capsid protein, Co-expression

## Abstract

**Background:**

Viruses cause significant economic losses to shrimp aquaculture worldwide. In severe cases, they can lead to 100% mortality within a matter of days, hence the aquaculture industry requires antiviral strategies to minimize economic impacts. Currently, a double-stranded RNA (dsRNA)-based platform has been proven effective at a laboratory scale. The bottleneck for its industrialization is the lack of low-cost, efficient and practical delivery approaches. In an effort to bridge the gap between laboratory and farm applications, virus-like particles (VLP) have been used as nanocarriers of dsRNA. However, the implementation of this approach still suffers from high costs and a lengthy procedure, co-expression of subunits of VLP or capsid proteins (CPs) and dsRNA can be the solution for the problem. CP and dsRNA are traditionally expressed in two different *E. coli* hosts: protease-deficient and RNase III-deficient strains. To condense the manufacturing of dsRNA-containing VLP, this study constructed a novel *E. coli* strain that is able to co-express viral capsid proteins and dsRNA in the same *E. coli* cell.

**Results:**

A novel bacterial strain DualX-B15(DE3) was engineered to be both protease- and RNase III-deficiency via P1 phage transduction. The results revealed that it could simultaneously express recombinant proteins and dsRNA.

**Conclusion:**

Co-expression of viral capsid proteins and dsRNA in the same cell has been shown to be feasible. Not only could this platform serve as a basis for future cost-effective and streamlined production of shrimp antiviral therapeutics, it may be applicable for other applications that requires co-expression of recombinant proteins and dsRNA.

**Supplementary Information:**

The online version contains supplementary material available at 10.1186/s12866-021-02148-8.

## Background

Viral pathogens have devastated global shrimp aquaculture for decades. It was assessed that shrimp production losses owing to viral diseases were around USD 15 billion over the last decade [1]. Accordingly, practical antiviral strategies are in urgent demand. However, as invertebrates, shrimp lack an adaptive immune system, rendering vaccination ineffective. As a result, many antiviral approaches that have been studied rely on enhancing host innate immunity [2]; examples include oral administration of probiotics [3], peptidoglycans [4] of β-glucan [5] and plant or seaweed extracts [6]. Other studies revealed that shrimp was capable of deploying an antiviral immune response via the RNA interference (RNAi) pathway [7, 8], as evidenced by the finding that injection of double-stranded RNA (dsRNA) constructs with sequences that matched viral mRNA was effective in preventing viral diseases or reducing their severity [9].

RNA interference (RNAi) is an endogenous gene-silencing mechanism that can be repurposed to combat viral infection through sequence-specific recognition and degradation of viral RNA [10]. As one of the most infamous shrimp viruses, RNAi applications against White Spot Syndrome Virus (WSSV) have been extensively investigated [11, 12] and dsRNA targeting the VP28 mRNA, which encodes the virus envelope protein that has been implicated in host entry [13, 14], has become one of the most effective targets [15]. Despite the promising results at a laboratory level, the implementation of the RNAi technology at a farm level is still at its infantile stage due to the lack of effective delivery approaches.

Many dsRNA-packaging strategies have been explored such as chitosans, liposomes and virus-like particles (VLP), which are self-assembled viral capsid proteins (CP) that are devoid of infectious nucleic acids. Among these, the VLP delivery systems have proved promising as their structures and attributes resemble native viruses. The advantages of VLP include the absence of infectious viral genetic materials, lack of infectivity, and specificity to the host cells [16]. Two types of VLP have, thus far, been applied for shrimp aquaculture: infectious hypodermal and hematopoietic necrosis virus (IHHNV) VLP and *Macrobrachium rosenbergii* nodavirus (MrNV) VLP. These VLPs are non-enveloped and composed of a single type of CP, which allows them to simply be repurposed as a nanocarrier.

In aquaculture, VLP provide potential delivery options because they can be recombinantly expressed and prolong the half-life of their dsRNA cargo by protecting them from nucleases as demonstrated in two previous reports [7, 17–19]. In the first example, it was revealed that dsRNA encapsulated in IHHNV-VLP conferred higher protection against YHV in *Penaeus vannamei* relative to naked dsRNA [19]. In the second example, the finding showed that delivery of dsRNA-VP28 encapsulated in MrNV-VLP gave protection against WSSV challenge in *P. monodon*: the survival rate of shrimp around 70% was observed in the encapsulated dsRNA treatment group, compared with 53 and 0% in the groups injected with naked dsRNA and phosphate buffer saline (PBS), respectively [7]. In addition to increasing the survival rate of shrimp, the MrNV-VLP itself elicited an innate response in shrimp by upregulation of a number of key immune genes [8].

The gridlock in the production of dsRNA-encapsulating VLP is the lengthy manufacturing process which includes individual expressions of CP and dsRNA in two separate bacterial hosts, followed by a complicated in vitro assembly to insert dsRNA into VLP. To reduce the production cost of the VLP-based delivery method, one possible scenario could be co-expression of VLP and dsRNA in a single culture. Currently, the *Escherichia coli* BL21(DE3) and HT115(DE3) strains are commonly used to independently express recombinant proteins and dsRNA, respectively. BL21(DE3) is deficient in two major protease-encoding genes but has an active dsRNA-specific RNase III-encoding gene. In contrast, HT115(DE3) [20] lacks the RNase III-encoding gene but contains protease-encoding genes.

This study, therefore, aimed to combine the strengths of these two strains of *E. coli* into a single platform that can simultaneously express recombinant proteins and dsRNA. The P1 phage system was employed to disrupt the RNase III-encoding gene in the protease-deficient BL21(DE3). To our best knowledge, this is the first attempt to engineer *E. coli* to be both protease- and RNase III-deficient for co-expression of recombinant proteins and dsRNA.

## Results

### Engineering of *E. coli* strain by the P1 phage system

**Fig. 1:**
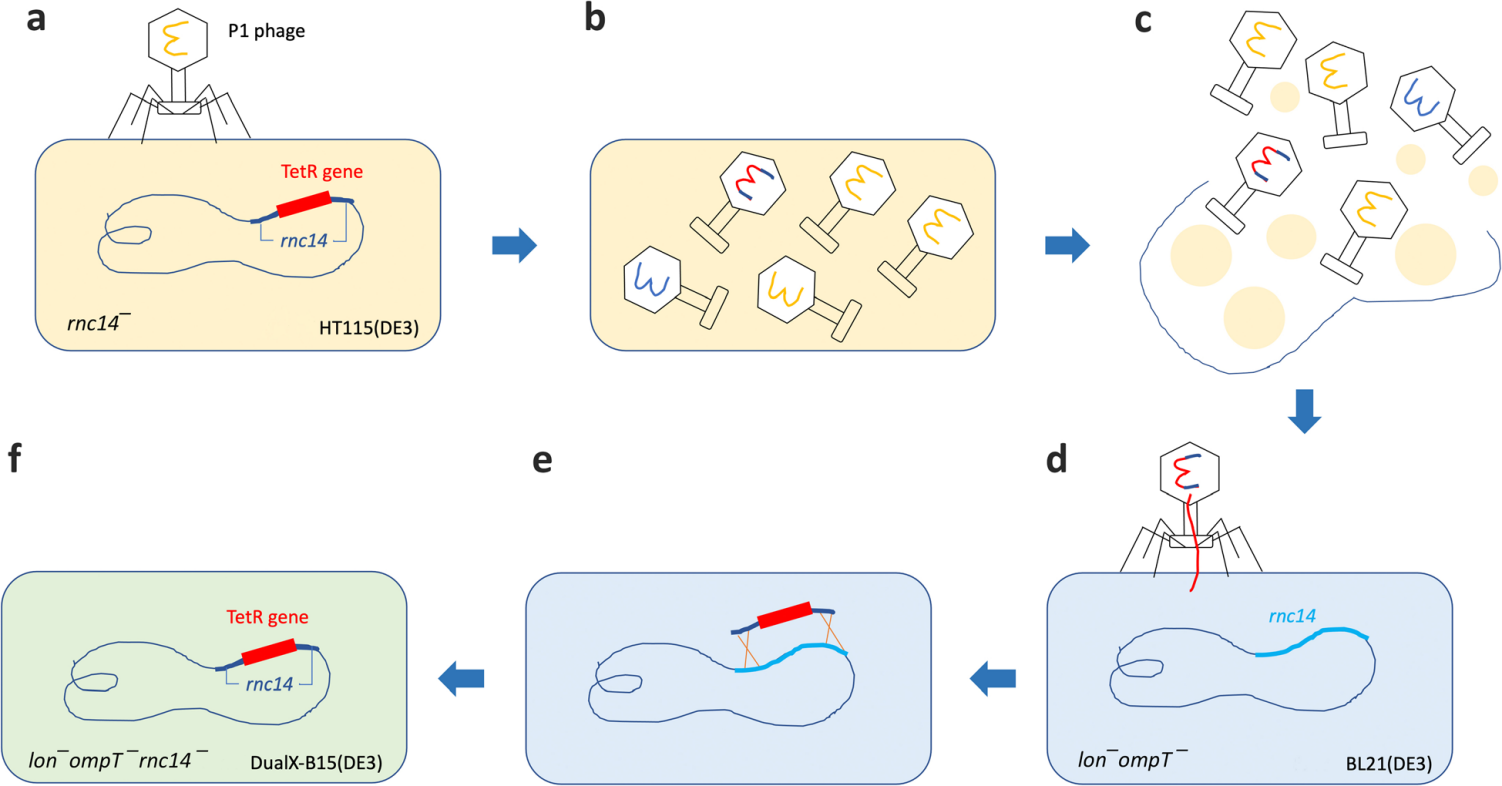
Construction of the RNase III- and protease-deficient DualX-B15(DE3) strain by P1 phage transduction. **a** Infection of the donor strain HT115(DE3) by P1 bacteriophage. **b** Random incorporation of the donor genome into viral capsids. **c** Release of new viral particles upon lysis of the donor cells. **d** Infection of the recipient strain BL21(DE3) with P1 lysate. **e** Homologous recombination at the *rnc14* gene locus. **f** Generation of the novel *E. coli* strain DualX-B15(DE3) with a tetracycline resistant marker (TetR)

Many gene knockout techniques had been considered. At the end, however, P1 transduction was chosen due to its technical simplicity. The technique exploits the DNA packaging mechanism of P1 bacteriophage. Briefly, during growth and packaging of its own chromosome, P1 bacteriophage occasionally encapsidates DNA from a donor bacterial strain into its capsid, and as a result some of the new phage particles contain donor’s DNA [21] (Fig. 1a and b). Upon infecting a recipient bacterial strain with the P1 donor lysate, the host homologous recombination machinery recombines and integrates the donor-derived DNA pieces into the recipient’s chromosome [22] (Fig. 1d and e). Through this “hitchhiking” mechanism, any DNA sequences from the donor bacterial strain can be transferred to the recipient strain, provided that the cassette-flanking regions and the target site are homologous.

There are two possible strategies for generating a new bacterial strain that is both protease- and RNase III-deficient. First, the protease-deficient BL21(DE3) could be rendered RNase III-deficient by mutating the RNase III-encoding gene, *rnc14*, through a single P1 phage transduction step. Alternatively, at least two P1 phage transduction events would be necessary to eliminate *lon* and *ompT* gene activities from the RNase III-deficient HT115(DE3). Therefore, the first approach was utilized in this study due to its convenience.

To knockout the *rnc14* gene from the recipient strain BL21(DE3), HT115(DE3) was used as a donor strain. The *rnc14* gene of HT115(DE3) is disrupted by Transposon 10 (Tn10) which harbors a tetracycline resistant cassette (TetR). To generate P1 lysate from the HT115(DE3) donor strain, P1 bacteriophage was allowed to infect HT115(DE3), so that they can randomly capture fragments of the HT115 genome, including parts of the Tn10-containing *rnc14* gene. Upon infecting the recipient BL21(DE3) with the P1 lysate, homologous recombination will occur between the recipient *rnc14* gene and its phage-derived counterpart, concomitantly resulting in the introduction of the tetracycline resistant cassette into the recipient genome and the disruption of the *rnc14* gene in the BL21(DE3) recipient genome (Fig. 1).

### Selection of the novel *E. coli* strain

A successful gene knockout was detected by tetracycline resistance acquired by the recipient strain BL21(DE3) during transduction. BL21(DE3) treated with the P1 donor lysate was plated on LB agar plates with or without tetracycline. The growth of colonies on tetracycline-supplemented plates was used as an indicator for desirable recombination that resulted in disruption of the *rnc14* gene. By comparing the numbers of colonies on the tetracycline-containing and control plates, the efficiency of selection was determined to be 0.1–1%.

**Fig. 2:**
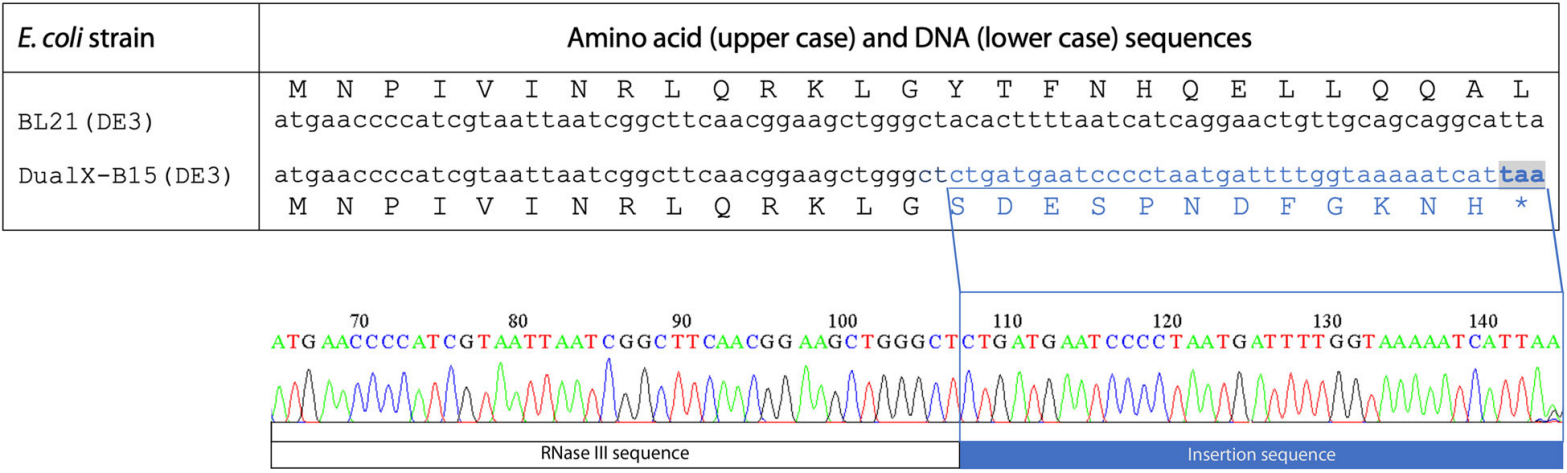
*E. coli* DualX-B15(DE3) was generated by incorporation of a premature stop codon into the *rnc14* gene of the donor strain BL21(DE3). Alignments of the amino acid and nucleotide sequences of the *rnc14* gene from the BL21(DE3) (Top) and DualX-B15(DE3) (Bottom) strains from the start codon (ATG). The asterisk at the end of the sequence of DualX-B15(DE3) indicates the premature stop codon (TAA). The nucleotide sequence inserted into the BL21(DE3) genome during P1 phage transduction is shown as blue underlined letters. The chromatogram from Sanger sequencing in the panel below showed the insertion mutation and the premature stop codon in the *rnc14* gene

To determine whether the *rnc14* gene in the tetracycline-resistant colonies contained any mutation that resulted in loss of function of the *rnc14* gene, chromosomal DNA of these colonies were extracted and sent for sequencing using primers that flanked the *rnc14* gene. The sequencing result showed that there was an insertion after the 14th codon of the *rnc14* gene. This insert coded for 12 amino acids followed by a premature stop codon which shortened the resulting protein from 226 amino acids to 26 amino acids (Fig. 2). The newly engineered strain was dubbed DualX-B15(DE3).

### Effect of *rnc14* knockout on bacterial growth

**Fig. 3:**
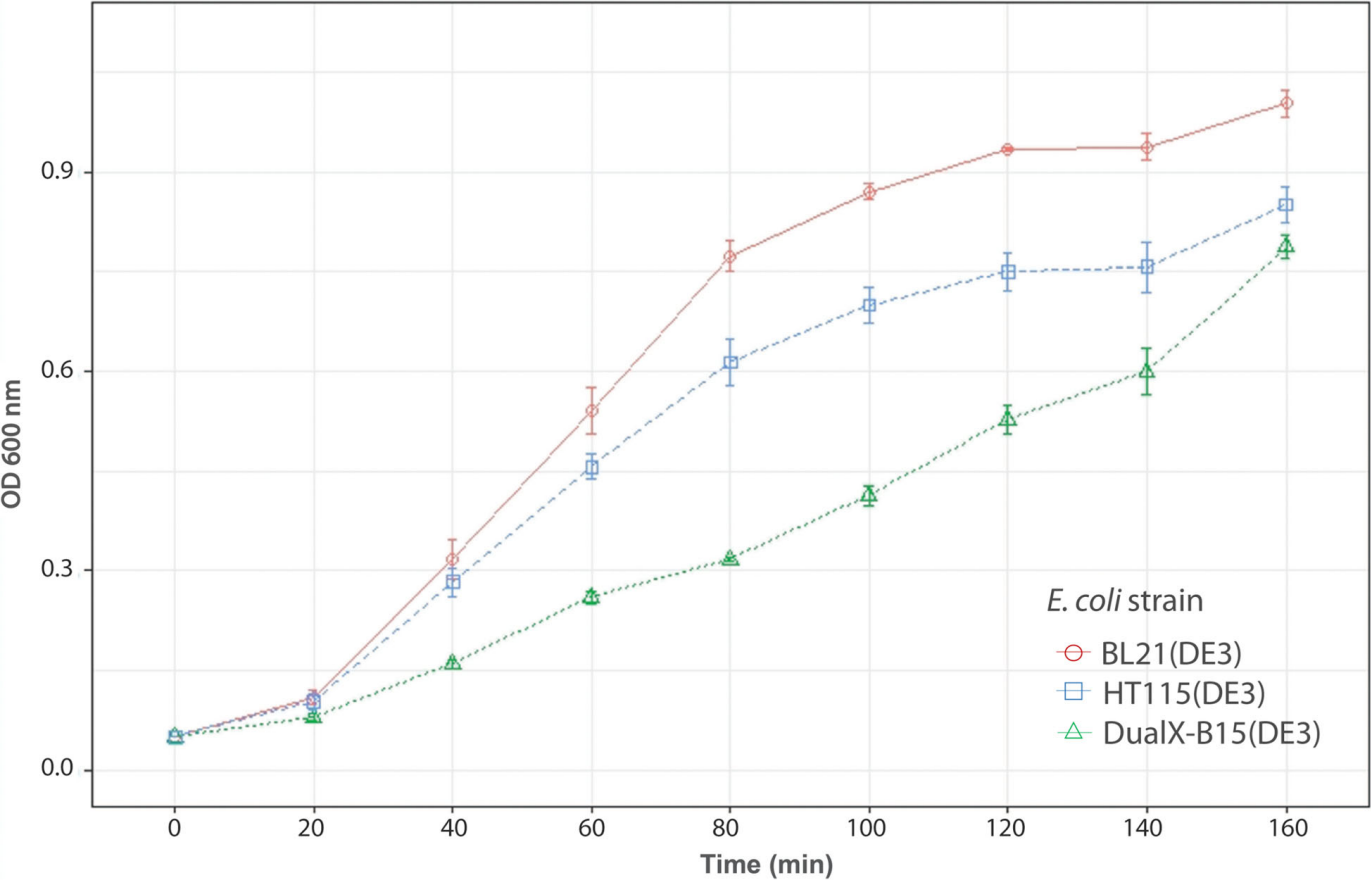
Knocking out the *rnc14* slowed down the growth rate of the newly engineered strain. Growth curve of BL21(DE3), HT115(DE3) and DualX-B15(DE3) are represented in red, blue and green, respectively

To investigate the effect of eliminating the *rnc14* gene activity in the novel *E. coli* strain on bacterial growth, BL21(DE3), HT115(DE3) and DualX-B15(DE3) were transformed with a dsRNA-expression plasmid. The growth of transformants at 37 °C was monitored by measuring optical density (OD) at 600 nm. It was shown that the growth rate of BL21(DE3) was highest, whereas the engineered strain exhibited the slowest growth rate and did not reach the stationary phase by the end of the monitored period (160 min) (Fig. 3).

### Expression of dsRNA in DualX-B15(DE3)

To demonstrate that DualX-B15(DE3) could indeed express dsRNA, expression of dsRNA-GW182 was conducted as a control as this dsRNA had been successfully expressed in our laboratory [23]. After dsRNA expression, the same wet mass of each bacteria culture was used to extract RNA, which was then subjected to an RNase-digestion assay and agarose gel electrophoresis to verify the double-stranded nature of the extracted RNA.

**Fig. 4:**
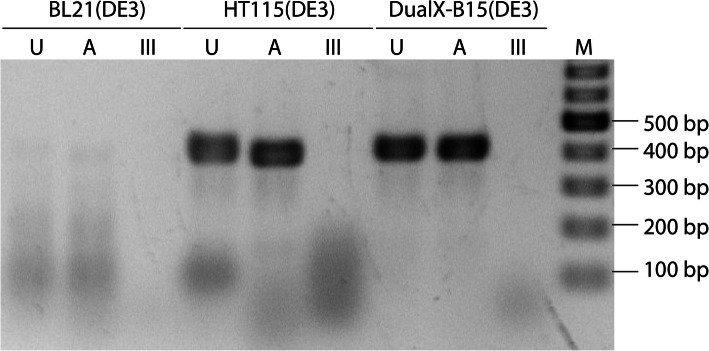
Double-stranded RNA could be expressed in DualX-B15(DE3). RNA analyzed on the gel included untreated samples (U), samples treated with RNase A (A) and samples treated with RNase III (III). Lane M (Marker) is a 2-log DNA ladder. The dsRNA were extracted from 50 mg wet weight of bacteria

As a positive control, the untreated sample from HT115(DE3) showed a discrete band at 454 bp (Fig. 4). When the same RNA sample was incubated with RNase A, which specifically cleaves single-stranded RNA, the band migrated faster as the loop region of the dsRNA hairpin was hydrolyzed by the enzyme. When the same RNA was treated with dsRNA-specific RNase III, no discrete RNA band was observable. Instead, a dark smear which is indicative of degraded nucleic acids was clearly evident at low molecular weight. Altogether, the three reactions verified that the RNA purified from HT115(DE3) was double-stranded in character. In contrast, no RNA from BL21(DE3) survived the enzymatic treatments, as only dark smears were found in the lanes from those samples. The analysis of RNA samples purified from DualX-B15(DE3) was identical to that of the dsRNA-producing HT115(DE3). Hence, it can be concluded that the newly engineered strain could indeed express dsRNA.

To compare the dsRNA-GW182 yields, three bacteria cultures were grown, and RNA was purified from identical wet cell mass. It was revealed that the dsRNA yields for this particular dsRNA construct were 0.92 ± 0.07 μg/mg wet cell mass for HT115(DE3) and 1.63 ± 0.22 μg/mg wet cell mass for DualX-B15(DE3).

### Expression of recombinant proteins in DualX-B15(DE3)

**Fig. 5:**
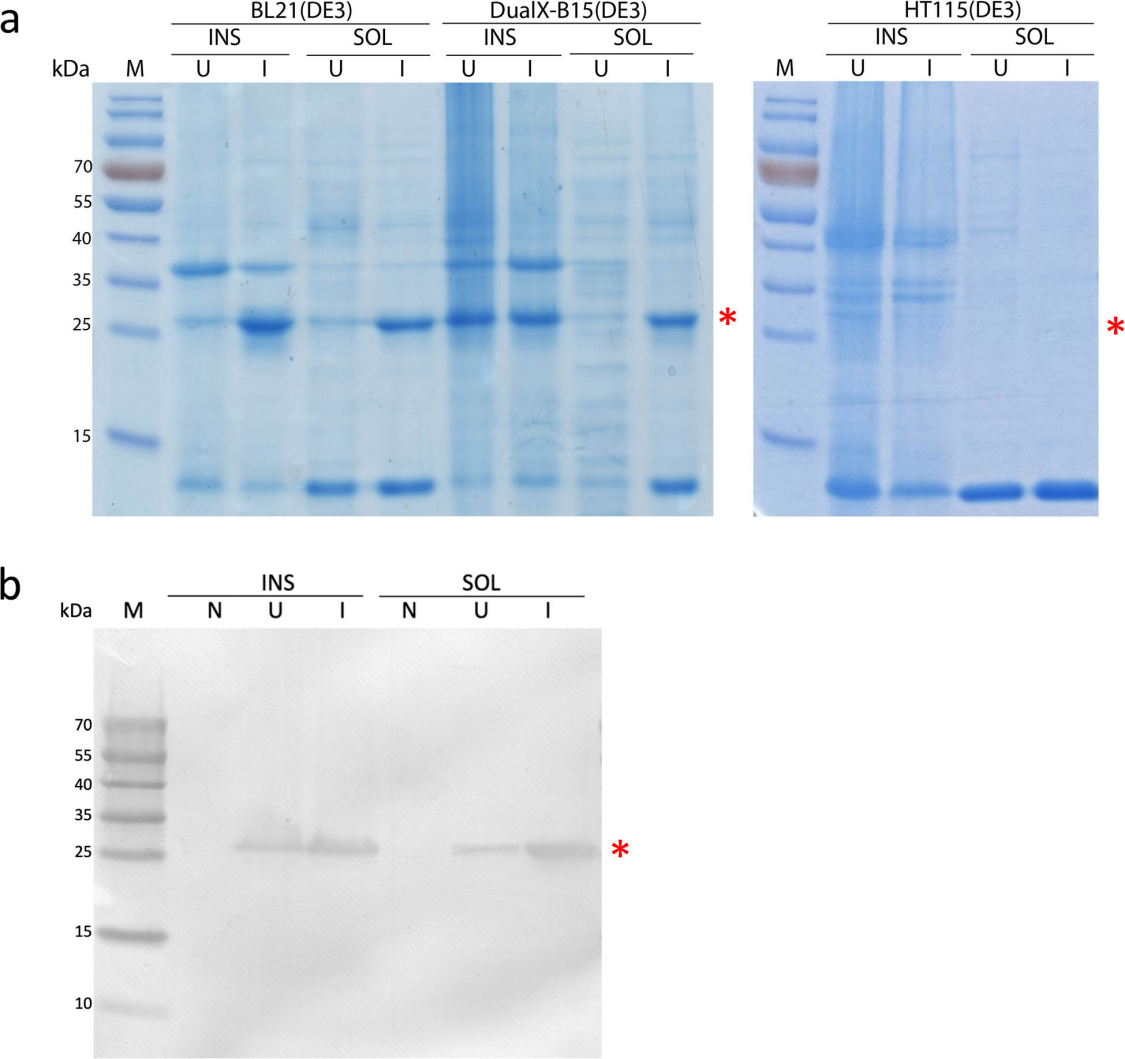
Recombinant proteins could be expressed in DualX-B15(DE3). **a** SDS-PAGE analysis of GST expressed in HT115(DE3), BL21(DE3) and DualX-B15(DE3) strains. **b** Western blot of GST expressed in DualX-B15(DE3). Lane M: prestained protein marker; Lane N: cells without the protein-expression plasmid; Lane U: uninduced cells with the protein-expression plasmid; Lane I: induced cells; Lane INS: insoluble fraction; Lane SOL: soluble fraction. The molecular weight of GST is 26 kDa as indicated by a red asterisk

To determine whether the newly engineered *E. coli* strain can express proteins, Glutathione S-Transferase (GST) was used as a control because it can be readily overexpressed in many *E. coli* strains. To compare GST expression in three different strains of *E. coli*, the same wet weight of bacterial cells was used for protein analysis. The soluble 26-kDa GST protein was successfully overexpressed in BL21(DE3) and DualX-B15(DE3) as revealed by the presence of the overexpression band at the corresponding molecular weight (Fig. 5a). On the other hand, no equivalent band was detected in the sample from HT115(DE3).

To further confirm that the overexpressed protein band in DualX-B15(DE3) was GST, Western blot was performed using an antibody against GST (Fig. 5b). A band at 26 kDa was observed in both the soluble and insoluble fractions, suggesting that the band in question was indeed GST. Therefore, the protein expression capacity of the parental BL21(DE3) strain was retained in DualX-B15(DE3), uncompromised by the knockout of the *rnc14* gene.

To compare the yield of GST expressed from the three bacterial strains, the amount of GST on the SDS-PAGE was estimated by comparing to the intensities of the pre-stained protein markers to that of the GST bands. Both DualX-B15(DE3) and BL21(DE3) expressed approximately 14 μg GST/mg wet cell mass.

In the following co-expression study, bacterial cells without any expression plasmids were used to represent background expression instead of uninduced cells because a small amount of recombinant protein was detected prior to IPTG induction as shown in the Western blot (Fig. 5b).

### Co-expression of dsRNA-VP28 and MrNV capsid protein (MrNV-CP)

Having shown that the new bacterial strain could express dsRNA and recombinant proteins individually, the next step was to co-express the two biological molecules in the same host. BL21(DE3), HT115(DE3) and DualX-B15(DE3) were co-transformed with dsRNA-VP28 and MrNV-CP expressing plasmids and co-expression of the recombinant products was induced with IPTG at 25 °C.

**Fig. 6:**
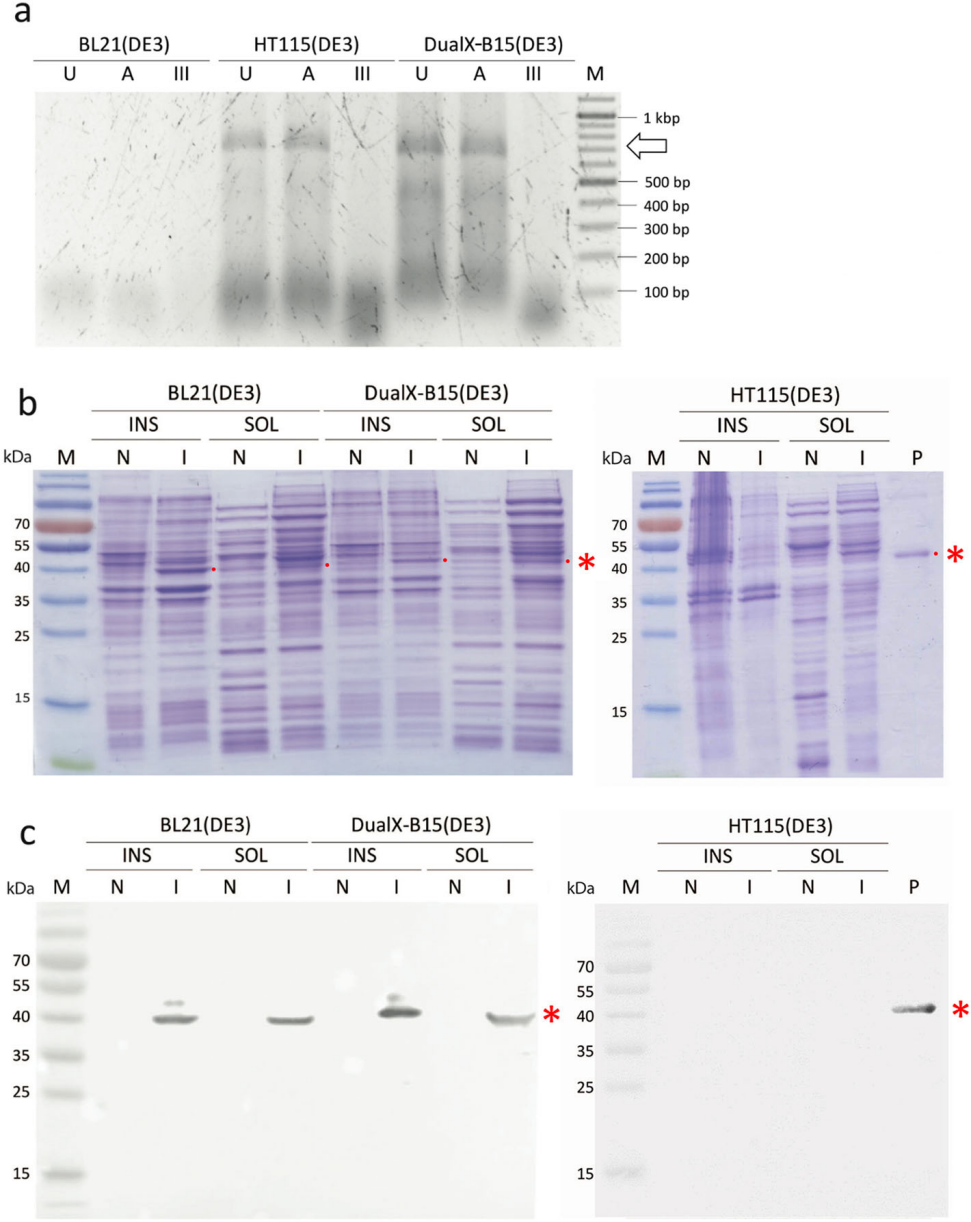
dsRNA-VP28 and MrNV-CP could be co-expressed in DualX-B15(DE3). a The RNase digestion assays: untreated (U), RNase A (A) and RNase III (III)-treated samples. Lane M (Marker) is a 2-log DNA ladder. The molecular weight of dsRNA-VP28 is indicated by an arrow. **b** SDS-PAGE analysis of protein expression in the three different strains of *E. coli*. A red dot is shown to the right of the expected band of MrNV-CP. **c** Western blot was performed with anti-MrNV capsid protein monoclonal antibody. Lane M: a pre-stained protein marker; Lane N: cells without the protein-expression plasmid; Lane I: induced cells; Lane P: a positive control (purified MrNV capsid proteins); Lane INS: insoluble fraction; Lane SOL: soluble fraction. The molecular weight of MrNV capsid protein is 40 kDa as indicated by a red asterisk.

Analysis of the resulting RNA revealed that both HT115(DE3) and DualX-B15(DE3) could produce dsRNA-VP28 at the expected size of 615 bp (Fig. 6a). In parallel, SDS-PAGE and Western blot in the presence of an anti-MrNV antibody confirmed the expression of MrNV-CP at 40 kDa in BL21(DE3) and DualX-B15(DE3) (Fig. 6b and c). Overall, the experiment showed that only DualX-B15(DE3) was capable of concurrent expression of both dsRNA-VP28 and MrNV-CP.

## Discussion

Previous reports have documented beneficial effects of VLP-encapsulated dsRNA over their unpackaged counterparts in controlling viral infection in shrimp [7, 8, 17, 19]. To increase the practicality of this platform, this study has successfully streamlined the co-expression of CP and dsRNA in the same cell by utilizing the P1 phage system to construct DualX-B15(DE3). The transduction process exhibited 0.1–1% efficiency which is considered typical according to a published report [24].

In this study, the sequence downstream from the premature stop codon that disrupted the *rnc14* gene in DualX-B15(DE3) was not further investigated once it was found that the newly engineered strain was able to express dsRNA while retaining its original protein expression capacity. In other words, the lack of the sequencing result did not alter the conclusion that the P1 transduction method knocked out the *rnc14* gene without altering the original protein expression genotype.

The fact that DualX-B15(DE3) could express both GST and MrNV-CP indicated that the protein expression capacity of the novel strain is not sequence-specific. While the amount of GST expression in DualX-B15(DE3) and BL21(DE3) appeared to be similar, further experiments are necessary to draw any conclusion about the quantitative nature of protein expression in these two strains.

When expressed in separate hosts, MrNV-VLP have to be disassembled into their capsid proteins (CP) building blocks in vitro in the presence of a chelating agent in order to introduce dsRNA and, then, reassembled upon the removal of the chelator [17]. We hypothesize that by synchronous co-expression of CP and dsRNA, dsRNA-containing VLP can be spontaneously formed based on a previous Cryo-EM report [25] which revealed that when MrNV-CP are expressed, they automatically form VLP that engulf random RNA from the expression host. Further studies may improve dsRNA-encapsulation specificity by introducing a pair of affinity tags into the N-terminus of CP, which protrudes inside the VLP, and the hairpin of the target dsRNA.

Even though DualX-B15(DE3) contains the desirable dsRNA and protein co-expression traits, it seemed that the removal of the *rnc14* gene activity via the P1 phage system resulted in the slow growth rate of the newly engineered strain. However, further optimization of growth conditions such as a change of media may curb this shortcoming. Despite this drawback of DualX-B15(DE3), its ability to co-express dsRNA-containing CP in vivo may outweigh its slower growth rate. In addition, based on the two dsRNA constructs tested in this study, it appeared that dsRNA yield in DualX-B15(DE3) was higher than HT115(DE3). However, whether this observation could be universally applied to other dsRNA constructs remained to be validated.

Late during the course of this study, it was revealed that insects do have an adaptive immune response that functions via the RNAi pathways [26]. Related research with shrimp suggests that they may be capable of a similar adaptive immune response. If this turns out to be correct, practical applications for the development of true vaccines in shrimp are likely to be possible and will be based on nucleic acid constructs rather than proteins. A key issue for successful applications would be the mode of preparation and effective delivery of the constructs via feeds. Thus, the results of this study may be even more significant than originally conceived.

## Conclusion

By using P1 phage transduction in which HT115(DE3) and BL21(DE3) were used as the donor and recipient strains, respectively, this study engineered a novel protease- and RNase III-deficient *E. coli* strain, DualX-B15(DE3) which can simultaneously express recombinant proteins and dsRNA.

## Methods

### Construction of recombinant plasmids

The pET17b-GST was constructed by using Glutathione S-Transferase encoding gene (Genbank accession number M97937.1). This gene was inserted into *Nde*I and *Sac*I restriction sites of pET17b plasmid. The pET28a-MrNV was synthesized by General Biosystems. To construct the plasmid, a gene encoding an MrNV capsid protein (Genbank accession number EU150129.1) with a C-terminus six-histidine was inserted between *Nco*I and *Xho*I restriction sites of an empty pET28a plasmid.

### Preparation of donor bacteriophage P1

To prepare P1 lysate from a donor strain**,** HT115(DE3) was grown overnight with shaking at 37 °C in 5 ml lysogeny broth (LB) with 12.5 μg/ml tetracycline. The overnight culture was diluted 100-fold with 4 ml antibiotic-free LB containing 5 mM CaCl_2_, 10 mM MgCl_2_ and 5 mM glucose. The culture was incubated at 37 °C for 1 h with shaking at 250 rpm. Then, 100 μl of *E. coli* bacteriophage P1 (ATCC® 25404­B1™) was added to the donor bacteria culture and incubated at 37 °C with aeration overnight. Afterwards, 50 μl of chloroform was added and mixed by vortex for 30 s. The mixture was centrifuged at 13,200×g for 1 min, before collecting the P1 phage-containing supernatant into a fresh tube.

### P1 phage transduction

To infect the recipient strain BL21(DE3) with the P1 lysate, BL21(DE3) was grown overnight at 37 °C in 5 ml antibiotic-free LB broth. Then, 2 ml of the culture was centrifuged at 4500×g. The BL21(DE3)-containing pellet was resuspended in 250 μl of LB broth containing 10 mM MgSO_4_ and 5 mM CaCl_2_. Next, 100 μl of the P1 lysate from the previous step was mixed with 100 μl of the resuspended recipient cells and incubated at 37 °C for 30 min on a bench. To chelate calcium and minimize secondary infection of P1 phage [27], 1 ml of LB media containing 0.2 M citrate was added. The mixture was further incubated with shaking at 250 rpm for 1 h at 37 °C, before being centrifuged at 4500×g for 1 min. The resulting sediment was resuspended in 100 μl of a fresh LB media containing 0.2 M citrate. The suspension was equally divided and plated on LB agar plates with and without 12.5 μg/ml tetracycline.

### Selection and verification of desirable mutants

Colonies grown on the tetracycline-supplemented plates were selected for further chromosomal DNA extraction by Qiagen genomic DNA extraction kit. The resulting DNA was used as a template DNA to amplify the *rnc14* gene by polymerase chain reaction (PCR) using an rnc-KO forward primer (5′-AAA CTG CAG CGA AGC AGT TA-3′) and an rnc-KO reverse primer (5′-TCA TTC CAG CTC CAG TTT TT-3′). The primer sequences were designed based on the *E. coli* str. K-12 substr. MG1655 genome (GenBank accession number NC_000913.3). The forward primer annealed upstream from the start codon between nucleotide positions 2,704,131 and 2,704,150. The reverse primer annealed adjacent to the stop codon between the nucleotide positions 2,703,383 and 2,703,402. A 50 μl PCR reaction mixture contained a final concentration of 1x reaction buffer, 1.5 mM MgCl_2_, 0.2 mM dNTPs, 0.5 μM of each forward and reverse primers, 100 ng extracted DNA, and 1 unit of Taq DNA polymerase (Invitrogen). Thermocycling conditions were at 94 °C for 3 min; followed by 30 cycles of 94 °C for 45 s, 55 °C for 30 s, 72 °C for 1 min; then a final extension at 72 °C for 10 min. To identify a mutation, PCR amplicons from wild type and mutant bacteria were purified and sent for sequencing by using the rnc-KO forward and reverse primers (Macrogen).

### Determination of the gene knockout effect on bacterial growth

To test the effect of *rnc14* knockout on the growth of the novel *E. coli* strain, the pET28a-GW182 plasmid [23] was transformed into BL21(DE3), HT115(DE3) and DualX-B15(DE3) for simulation of the actual expression condition of double-stranded RNA (dsRNA). The transformants were grown in LB media containing 50 μg/ml kanamycin (for all strains) and 12.5 μg/ml tetracycline (for HT115(DE3) and DualX-B15(DE3)). The overnight cultures were diluted in 15 ml of fresh LB media supplemented with the corresponding antibiotics and OD_600_ was adjusted to 0.05. Then, they were incubated at 37 °C with shaking at 250 rpm. The growth of bacterial strains was observed by measuring OD_600_ every 20 min for 160 min by NanoDrop One Spectrophotometer (Thermo Fisher Scientific). Experiments were performed in triplicate to obtain average OD_600_ at each time point.

### Expression of dsRNA

To express dsRNA, the pET28a-GW182 plasmid [23] was transformed into BL21(DE3), HT115(DE3) and DualX-B15(DE3). The transformants were grown in LB media containing 50 μg/ml kanamycin (for all strains) and 12.5 μg/ml tetracycline (for the HT115(DE3) and DualX-B15(DE3)) overnight. The cultures were diluted 100-fold in 25 ml of antibiotic-supplemented fresh LB media and OD_600_ was adjusted to 0.1. Then, they were incubated at 37 °C with shaking at 250 rpm for 2 h until OD_600_ reached 0.4. To induce the expression of dsRNA, 1 mM IPTG was added to the cultures for 3 h at 37 °C before the final OD_600_ was measured by a spectrophotometer. The cultures were harvested by centrifugation at 4500×g for 5 min.

### Purification of dsRNA

To extract dsRNA by the ethanol extraction method [28], 50 mg of the resulting wet bacterial pellet was resuspended with 1 ml of 75% ethanol in 1x PBS (Sigma-Aldrich) and incubated at − 20 °C overnight. The mixture was centrifuged at 6000×g for 5 min at 4 °C, resuspended with 0.3 ml of 150 mM NaCl, and incubated at room temperature for 1 h. To isolate the soluble fraction, the suspension was centrifuged at 8000×g for 10 min at 4 °C and dsRNA-containing supernatant was collected.

To compare the yields of dsRNA-GW182 among different *E. coli* strains, the bacteria culture was done in triplicate. To visualize the extracted RNA, RNA isolates were analyzed by 1.5% agarose gel electrophoresis. Analysis of RNA band intensity was performed using ImageJ. The concentration of dsRNA was calculated by comparing the band intensity with that of the corresponding band in a 2-log DNA marker (New England Biolabs).

### RNase treatment of the purified RNA

To verify that the RNA expressed in the engineered strain was in the form of dsRNA, the resulting RNA was divided into three equal parts. One fraction was mixed with 1x RNase A buffer and distilled water for the untreated condition. The other fraction was treated with 0.01 μg/μl RNase A enzyme (New England Biolabs) in 10 μl reaction mixture containing 1x RNase A buffer. The remaining fraction was treated with 1 unit of RNase III enzyme (New England Biolabs) in 10 μl reaction mixture containing 1x RNase III buffer and 1x MnCl_2_. The reactions were incubated at 37 °C for 5 min before being analyzed by 1.5% agarose gel electrophoresis alongside a 2-log DNA ladder staining with ethidium bromide and then visualized under UV lamp of Gel Documentation (Bio-Rad).

### Expression of GST protein

To compare protein expression capacity of the three *E. coli* strains, the pET17b-GST plasmid was transformed into HT115(DE3), BL21(DE3) and DualX-B15(DE3). The bacteria were grown in LB medium containing 100 μg/ml ampicillin (for all strains) and 12.5 μg/ml tetracycline (for HT115(DE3) and DualX-B15(DE3)) at 37 °C with shaking at 250 rpm until OD_600_ reached 0.6. Protein expression was induced with IPTG at a final concentration of 1 mM and cells were then incubated at 25, 30 and 37 °C overnight. After centrifugation at 4500×g for 5 min, 50 mg of wet bacterial pellet was resuspended with 1 ml of lysis buffer pH 8 (50 mM NaH_2_PO_4_, 150 mM NaCl and 10 mM imidazole). Sonication of the cell suspension was performed at 40% amplitude with 2 cycles of 10 short bursts of 5 s followed by 5 s intervals for cooling (Sonics VCX 750). The lysates were then fractionated by centrifugation at 12,000×g. The total protein concentration was measured by using the Bradford reagent (Bio-Rad). The fractions were analyzed on 12.5% SDS-PAGE and Western blot using a mouse anti-GST antibody with a pre-stained protein ladder (Thermo Scientific).

To compare the GST yields among three *E. coli* strains, cell lysates were analyzed by SDS-PAGE to detect protein band. Analysis of protein band intensity was performed using ImageJ. The concentration of GST was calculated by comparing the band intensity with that of the corresponding band in 4 μl pre-stained protein ladder (Thermo Scientific).

### Co-expression of dsRNA and CP

To co-express dsRNA-VP28 and MrNV-CP in the three bacterial strains, HT115(DE3), BL21(DE3) and DualX-B15(DE3) were co-transformed with the pGEM-VP28 [7] and pET28a-MrNV plasmids for dsRNA and CP expression, respectively. The expression temperatures were varied at 25, 30 and 37 °C. The optimized condition of co-expression was found to be at 25 °C overnight because, while dsRNA-VP28 could be expressed at all of the above temperature (Fig. S1), the best expression temperature of MrNV-CP was 25 °C as previously reported in Jariyapong *et. al,* 2015 [7]. The bacterial cells were grown in LB medium containing 50 μg/ml ampicillin, 25 μg/ml kanamycin (for all strains) and 6.25 μg/ml tetracycline (for HT115(DE3) and DualX-B15(DE3)) at 37 °C with shaking at 250 rpm until OD_600_ reached 0.6. Co-expression of dsRNA and CP was induced with IPTG at a final concentration of 1 mM and cells were then incubated at 25 °C overnight before harvest by centrifugation at 4500×g for 5 min.

The cell wet weight used for dsRNA and CP extraction was 100 mg. Half of the bacteria culture was used for protein analysis, while the other half of culture was subjected to dsRNA purification and RNase treatment (see above). To analyze protein expression, the total amount of 7.5 μg total protein determined by Bradford assay was loaded in each lane of gel electrophoresis. The MrNV-CP expression was confirmed by Western blot. For dsRNA-VP28 analysis, the total amount of 2 μg RNA, as determined by a spectrophotometer, was loaded in agarose gel electrophoresis.

### Western blot

After SDS-PAGE, proteins were transferred onto a polyvinylidene fluoride (PVDF) membrane by Pierce Power Blotter (Thermo Scientific) for Western blot according to the manufacturer’s protocol. The membranes were immersed in 5% skim milk in PBS to block non-specific antibody binding at room temperature for 1 h with constant shaking. For GST protein detection, the membranes were immersed in a 1:1000 dilution of a mouse anti-GST antibody (Bio-Rad). For MrNV-CP expression, the membranes were immersed in a 1:100 dilution of an anti-MrNV-CP monoclonal antibody [17]. The membranes were washed three times for 5 min each with PBS containing 0.05% Tween-20 at room temperature with shaking. A secondary antibody (horseradish peroxidase conjugated goat anti-mouse IgG) was added at 1:2500 dilution and incubated for 1 h at room temperature with agitation. The membranes were washed three times with PBS containing 0.05% Tween-20. The antigen-antibody reactivity was detected by chemiluminescent method using Clarity Western ECL substrate (Bio-Rad).

## Supplementary Information

**Additional file 1: Figure S1.** dsRNA-VP28 could be expressed at 25 °C, 30 °C and 37 °C. RNA isolates were extracted from *E. coli* DualX-B15(DE3). RNA analyzed on the gel included untreated samples (U), samples treated with RNase A (A) and samples treated with RNase III (III). Lane M (Marker) is a 2-log DNA ladder. The molecular weight of dsRNA-VP28 was 615 bp as indicated by a triangle.

## Data Availability

All data generated or analyzed during this study are included in this published article.

## Abbreviations

dsRNA: Double-stranded RNA
CP: Capsid protein
VLP: Virus-like particles
RNAi: RNA interference
GST: Glutathione S-Transferase
WSSV: White spot syndrome virus
MrNV: *Macrobrachium rosenbergii* nodavirus
IHHNV: Infectious hypodermal and hematopoietic necrosis virus
