# Supplementary material for: Co-expression of double-stranded RNA and viral capsid protein in the novel engineered Escherichia coli DualX-B15(DE3) strain
Source: BMC Microbiol. 2021 Mar 23;21:88. doi: 10.1186/s12866-021-02148-8 (PMC7989029; doi:10.1186/s12866-021-02148-8)
Supplement: Supplementary file 1 — Additional file 1: Figure S1. dsRNA-VP28 could be expressed at 25 °C, 30 °C and 37 °C. RNA isolates were extracted from E. coli DualX-B15(DE3). RNA analyzed on the gel included untreated samples (U), samples treated with RNase A (A) and samples treated with RNase III (III). Lane M (Marker) is a 2-log DNA ladder. The molecular weight of dsRNA-VP28 was 615 bp as indicated by a triangle. [file 12866_2021_2148_MOESM1_ESM.docx]

**Supplementary information**


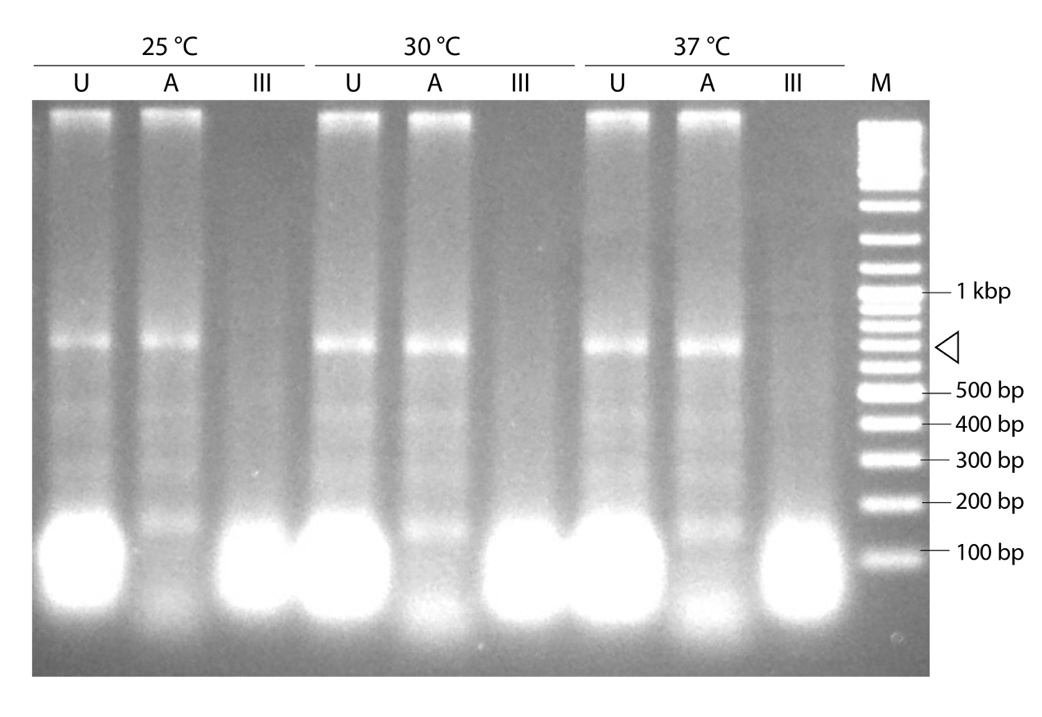


**Figure S1**. dsRNA-VP28 could be expressed at 25 °C, 30 °C and 37 °C. RNA isolates were extracted from *E. coli* DualX-B15(DE3). RNA analyzed on the gel included untreated samples (U), samples treated with RNase A (A) and samples treated with RNase III (III). Lane M (Marker) is a 2-log DNA ladder. The molecular weight of dsRNA-VP28 was 615 bp as indicated by a triangle.
